# Imidazole Post‐Treated Self‐Assembled Monolayers for Inverted Perovskite Solar Cells

**DOI:** 10.1002/advs.202518676

**Published:** 2025-12-26

**Authors:** Ihtesham Ghani, Shi Tingshu, Shehzad Ahmed, Zhuo Hongbin, Zhu Zirun, Yu Zhang, Peng You, Imran Muhammad, Tang Zeguo, Danish Khan

**Affiliations:** ^1^ College of Applied Technology, College of Physics and Optoelectronic Engineering Shenzhen University Shenzhen P. R. China; ^2^ Shenzhen Key Laboratory of Ultraintense Laser and Advanced Material Technology Center for Advanced Material Diagnostic Technology and College of Engineering Physics Shenzhen Technology University Shenzhen P. R. China; ^3^ The College of New Materials and New Energies Shenzhen Technology University Shenzhen P. R. China; ^4^ China‐UK Low Carbon College Shanghai Jiao Tong University Shanghai P. R. China; ^5^ National Industry‐Education Platform of Energy Storage Tianjin University Tianjin P. R. China

**Keywords:** inverted perovskite solar cells, post‐treatment, self‐assembled monolayers

## Abstract

Inverted perovskite solar cells have emerged as promising candidates for next‐generation photovoltaics due to their compatibility with tandem architectures and flexible substrates. A critical factor for high device performance is the optimization of buried interfaces using self‐assembled monolayers (SAMs), with Me‐4PACz standing out for its excellent charge extraction properties. However, a polarity mismatch between the hydrophobic carbazole terminal and the polar perovskite precursors hinders film coverage and efficient device reproducibility. Here, we report a facile post‐treatment strategy employing two chlorinated imidazole derivatives, 4,5‐dichloroimidazole (4,5‐DI) and 4,5‐dichloro‐2‐methylimidazole (4,5‐D‐2‐MI), at the Me‐4PACz/perovskite interface. These molecules enhance carbazole–imidazole interactions, convert the surface from nonpolar to polar, and improve the wettability of the SAM, resulting in an enhanced perovskite morphology. The resulting interfacial dipole modifications alter the work function and reduce the band offset at Me‐4PACz/perovskite interface, ultimately enhancing the device fill factor and photovoltage. Ultimately, the target devices delivered an efficiency of approximately 25% with improved long‐term stability under varied environmental conditions, highlighting the effectiveness of interfacial engineering via SAM post‐treatment for high‐performance and durable devices.

## Graphical abstract

A post‐treatment strategy using imidazole derivatives (4,5‐DI and 4,5‐D‐2‐MI) enhances the Me‐4PACz/perovskite interface by improving wettability, promoting perovskite films, and optimizing energy level alignment, resulting in higher efficiency and improved stability in inverted perovskite solar cells.

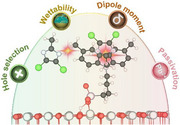

## 1 Introduction

Inverted perovskite solar cells (iPSCs) have gained significant attention due to their compatibility with tandem architectures and flexible substrates [1, 2, 3], positioning them as a promising candidate for next‐generation photovoltaics. A critical factor in their performance enhancement is the optimization of buried interfaces using self‐assembled monolayers (SAMs) [4, 5], with Me‐4PACz—a carbazole‐based phosphonic acid‐anchored SAM—emerging as a leading material due to its superior charge extraction properties [6]. However, challenges such as poor perovskite film coverage and non‐uniform SAM adsorption on substrates limit device reproducibility [7, 8, 9]. These issues primarily arise from weak chemisorption of phosphonic acid groups on metal oxide surfaces and a polarity mismatch between the hydrophobic carbazole terminal and polar perovskite precursor solvents, which disrupts uniform perovskite crystallization [10, 11, 12, 13, 14]. Additionally, the lack of strong passivation at the SAM/perovskite interface leads to defects, particularly undercoordinated Pb^2^⁺ ions [15, 16], further degrading device performance. Significant research efforts have been directed toward addressing these interfacial challenges through multiple innovative strategies. One approach involves the co‐assembly of Me‐4PACz with complementary headgroup molecules, which can improve SAM uniformity and enhance charge transport properties [17, 18, 19]. Another key strategy focuses on substrate surface treatments, such as plasma or chemical activation, to increase the density of chemisorption sites and enhance the anchoring of SAMs [20]. Researchers have also explored solvent engineering techniques to optimize the polarity matching between perovskite precursors and SAM‐modified surfaces, thereby promoting more homogeneous nucleation and growth of perovskite [21]. Additionally, substantial progress has been made in developing novel head groups based on carbazole derivatives decorated with polar chemistries [22, 23, 24, 25]. More specifically, carbazole is non‐polar, and ─CH_3_ is a hydrophobic functional group, which makes Me‐4PACz less compatible with the perovskite's precursor solvents.

Nevertheless, as an alternative and facile approach, recent studies have explored the post‐treatment of SAM to enhance the SAM's wettability and modify its interfacial properties. More specifically, the carbazole head of Me‐4PACz is covered by some polar molecule, thus enhancing the surface energy of the SAM layer. For instance, He et al. introduced p‐xylylenediphosphonic acid (p‐XPA) that has polar ─PO_3_H_2_ groups [16]. Similarly, Pitaro et al. deposited (4CzNH_3_I), an ionic liquid, on the carbazole‐based SAM to promote its wetting property [26]. In another study, 2‐(Diphenylphosphino)acetic acid (2DPAA) is deposited to improve the interfacial properties of the Me‐4PACz/perovskite interface [27]. Zhao et al. treated Me‐4PACz with (2‐bromoethyl)phosphonic acid (Br‐EPA) to enhance surface hydrophilicity [28]. However, selecting a suitable molecule for post‐treating Me‐4PACz is crucial—not only to enhance surface wettability but also to strengthen its interaction with the carbazole backbone, which serves as the primary electron‐rich *π*‐conjugated unit responsible for charge transport and interfacial electronic coupling with the perovskite. Because the orientation, packing, and intermolecular interactions of the carbazole moiety directly determine the quality of the buried interface, molecules capable of interacting favorably with this unit are essential for achieving optimized energy levels and improved device performance.

Herein, we applied a similar post‐treatment strategy using two imidazole derivatives, 4,5‐dichloroimidazole (4,5‐DI) and 4,5‐dichloro‐2‐methylimidazole (4,5‐D‐2‐MI), at the Me‐4PACz/perovskite interface. The imidazole ring introduces a unique polarity via its ─CN and ─NH moieties. Further, these chlorinated and conjugated imidazoles enhance interactions between the carbazole of Me‐4PACz and the imidazole ring via π‐π interactions or hydrogen bonds and convert the non‐polar behavior of the carbazole moiety of Me‐4PACz to polar. Thus enhancing the SAM's wettability and improving the perovskite morphological properties. Additionally, the enhanced dipole moments, shift in the work function, and reduction of the band offset between the hole‐transporting layer (HTL) and perovskite improve the fill factor (*FF*) and open‐circuit voltage (*V_OC_*) of the target devices. Thus, post‐treatment of Me‐4PACz with 4,5‐D‐2‐MI resulted in an efficiency of 24.97%, while the 4,5‐DI‐based device achieved an efficiency of 24.48%. Furthermore, the devices demonstrated improved long‐term stability under various environmental conditions due to the enhancement of the interfacial buried contact.

## 2 Results and Discussions

**FIGURE 1:**
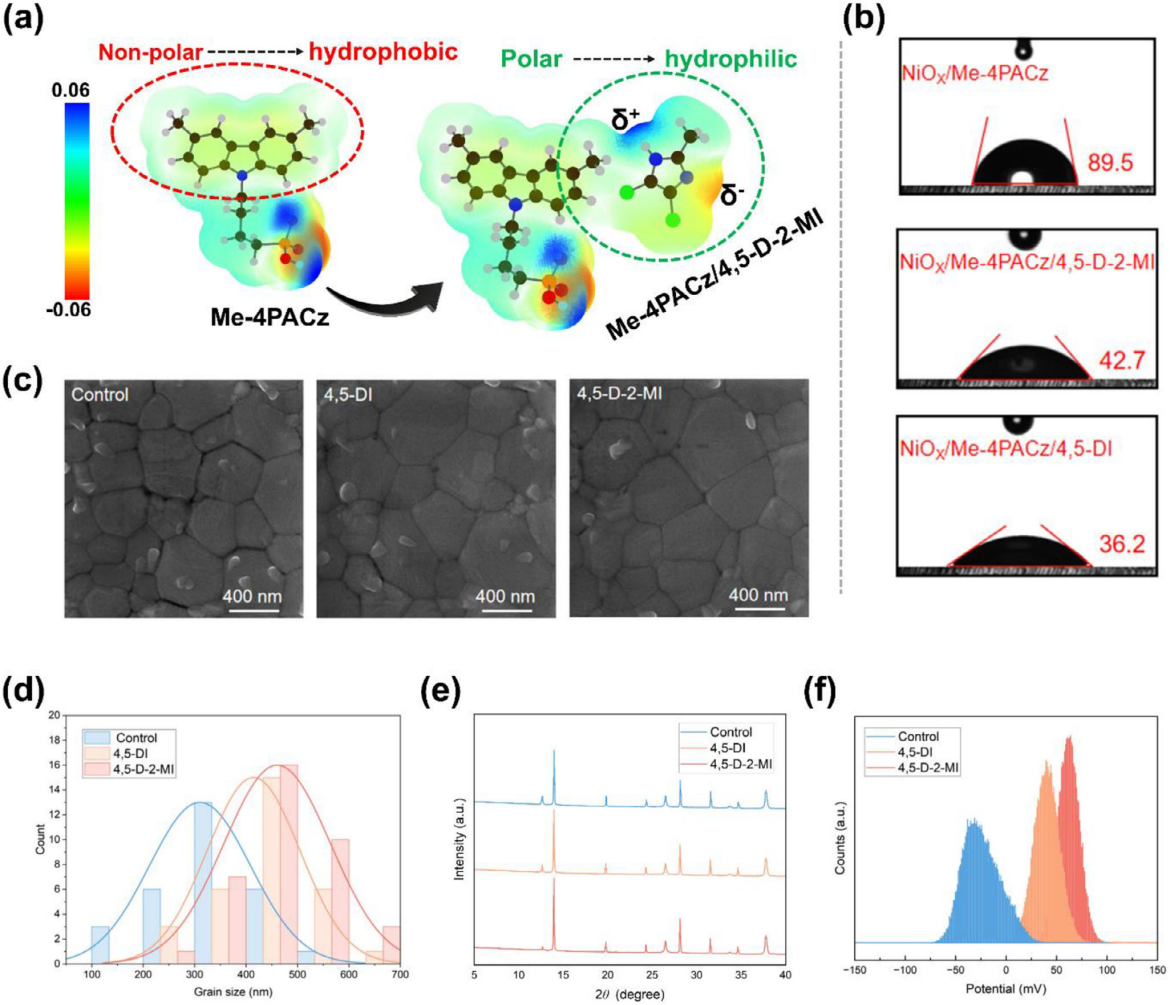
(a) ESPs of Me‐4PACz and Me‐4PACz/4,5‐D‐2‐MI. (b) water contact angles of NiO_X_/Me‐4PACz [Control], Me‐4PACz/4,5‐D‐2‐MI, and Me‐4PACz/4,5‐DI. (c) SEM images, (d) average grain sizes, (e) XRD analysis, and (f) KPFM potential counts of control (without interface modification) perovskite films and SAM post‐treatment strategy‐based perovskite films.

The chemical structures of both imidazole derivatives are provided in Figure S1. The conventional SAM is modified by post‐depositing 4,5‐DI or 4,5‐D‐2‐MI with an optimized concentration on NiO_X_/Me‐4PACz which enhances the wettability and polarity of the SAM layer as schematically presented in Figure 1a. The commonly used precursor solvent, dimethyl sulfoxide (DMSO), is a polar aprotic solvent which features a strongly electrostatic potential (ESP)‐negative sulfoxide oxygen (δ−), a partially positive sulfur (δ+), and two relatively less polar ─CH_3_ groups (Figure S2). Therefore, it loves to interact with positively polarized (δ+) groups. Nevertheless, the self‐assembled Me‐4PACz exposed part, a ─CH_3_‐decorated carbazole, presents a low molecular dipole moment (∼1.4–1.5 D) and weak electrostatic potential (ESP). These values indicate substantially lower polarity compared with DMSO which has a high dipole moment of 3.96 D and a strongly negative ESP region around the sulfoxide oxygen. In contrast, 4,5‐DI and 4,5‐D‐2‐MI contain –NH_2_ groups that exhibit positively polarized ESP regions and higher molecular dipole moments (2.5–2.9 D), leading to significantly stronger electrostatic complementarity with the negatively charged sulfoxide oxygen of DMSO.

These differences can be seen from the ESP mapping comparisons of Figure 1a. The film's hydrophilicity has been confirmed by comparing the contact angles in each case (Figure 1b), i.e., enhancements from 89.5° to 36.2° and 42.7° for 4,5‐DI and 4,5‐D‐2‐MI, respectively. As a result, the size enhancement and adhesion are observed in the grains of the modified films (Figure 1c,d) as well as the crystallinity, which can be seen from the comparison of the X‐ray Diffraction (XRD) patterns of the control with 4,5‐DI and 4,5‐D‐2‐MI‐based films (Figure 1e). Similar findings have been noticed in the cross‐sectional scanning electron microscopy (SEM) images (Figure S3). Modified films by both 4,5‐DI and 4,5‐D‐2‐MI enhanced the grain size and crystallinity of the perovskite film. Also, the film coverage of control was not as homogenized as target films (Figure S4). From the surface potential distributions obtained by KPFM for the control (NiO_x_/Me‐4PACz) and modified HTLs (Figure 1f; Figure S5), the modified HTLs exhibit both enhanced surface potential and improved uniformity, as illustrated in Figure 1f. The presence of chlorine and ─NH group could enhance the potential. The same scenerio is reflected by the theoritical ESP maps. Thus, along with the smoothness of potential, the NiO_X_’s root mean square (RMS) roughness values also became smoother after treatment (Figure S6).

**FIGURE 2:**
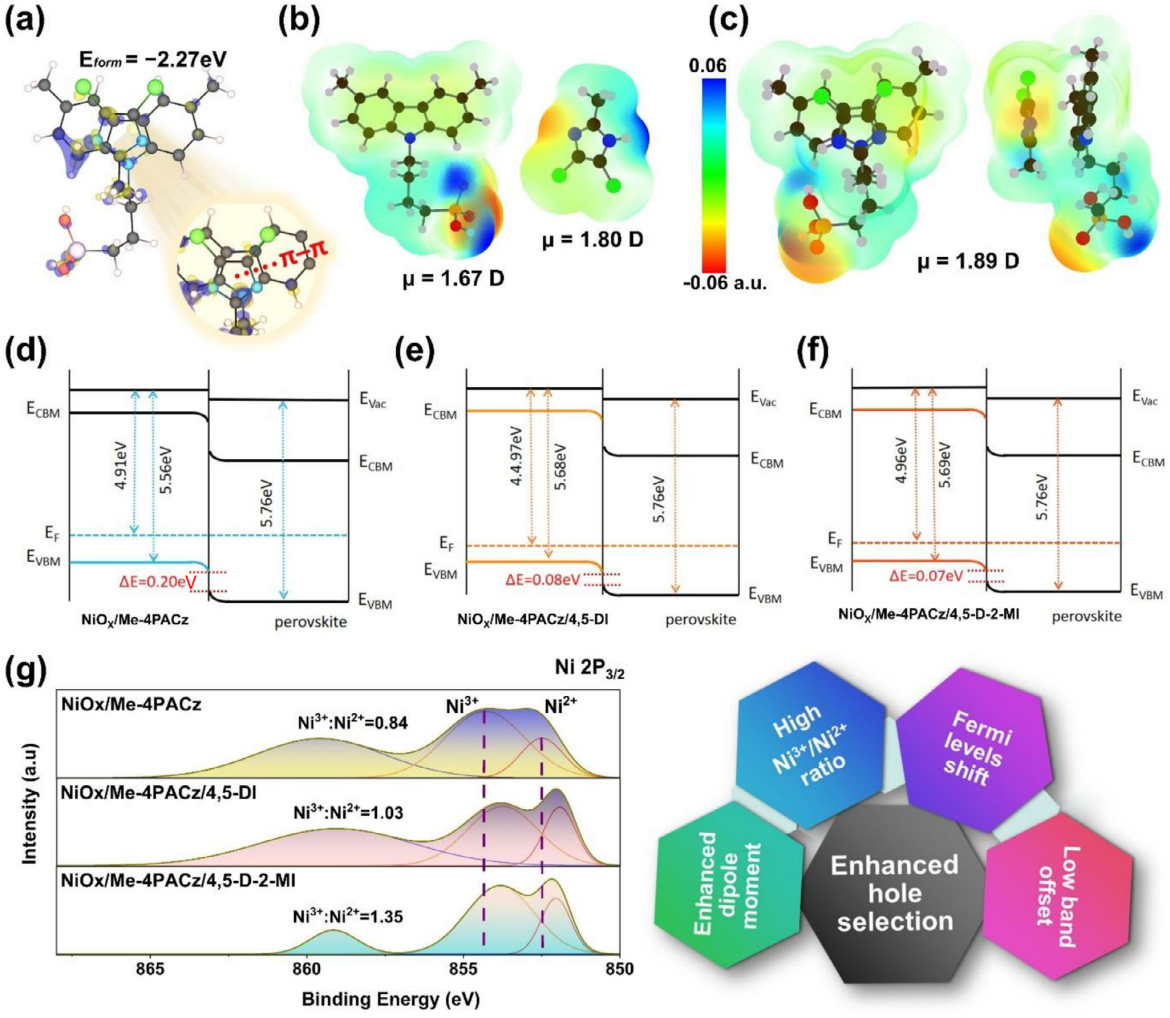
(a) Formation energy (*E_form_*) between the Me‐4PACz and 4,5‐D‐2‐MI. ESPs and dipole moments (µ) of (b) Me‐4PACz and Me‐4PACz:4,5‐D‐2‐MI and (c) Me‐4PACz:4,5‐D‐2‐MI system. (d–f) The energy levels and Fermi levels of NiO_X_/Me‐4PACz before and after 4,5‐D‐2‐MI and 4,5‐DI treatments. (g) The Ni 2P_3/2_ XPS spectra of NiO_X_/Me‐4PACz before and after 4,5‐D‐2‐MI and 4,5‐DI treatments.

To evaluate the interaction between the imidazole and Me‐4PACz, the density functional theory (DFT) calculations in two different scenarios: 4,5‐D‐2‐MI on the ─CH_3_ moiety of Me‐4PACz, and the *π*–*π* interaction between the conjugated carbazole and the imidazole ring have been performed. The calculated formation energies are provided in Figure S7a and Figure 2a, respectively. The *π*–*π* interaction had the highest formation energy of −2.27 eV (Figure 2a). A higher (more negative) formation energy indicates that the interaction is stronger and more stable, resulting in the molecule binding more tightly and influencing surface properties more significantly. Second, the chlorination of imidazole pulls the electron density from the imidazole ring, which makes the N─H proton more acidic, so the H‐bond donating ability increases. Therefore, a strong interaction, with the formation energy of −2.19 eV, has also been found in the CH_3_ moiety of Me‐4PACz and 4,5‐D‐2‐MI (Figure S7a). In any case, an enhanced dipole moment has been noticed (Figure 2c; Figure S7b). Even though the individual dipole moment of both Me‐4PACz and 4,5‐D‐2‐MI is 1.48 Debye (D) and 0.57 D, respectively. The combined dipole moment is high (about 1.89 D) because Me‐4PACz and 4,5‐D‐2‐MI not only align their intrinsic dipoles in the same direction on NiO_X_ but also engage in *π*–*π* interactions that stabilize this orientation leading to a reinforced net dipole which is beneficial for charge transportation at the interfaces [15, 29, 30]. To further validate the interaction, similar calculations have been done on the NiO_X_‐adsorbed Me‐4PACz, and an increased Me‐4PACz to 4,5‐D‐2‐MI interaction has been noticed again, with minimum differences in the formation energies of Me‐4PACz on NiO_X_ (Figure S8). The work functions, as determined from UV–vis and ultraviolet photoelectron spectroscopy (UPS) measurements (see Figure S9), showed an enhancement after 4,5‐DI or 4,5‐D‐2‐MI treatment (Figure 2d–f). These results are also aligned with KPFM findings. Similarly, the valence band maximum (VBM) shifted downward, and the band offset between the SAM and perovskite is reduced up to 0.07 eV. From the X‐ray photoelectron spectroscopy (XPS) analysis of Ni 2P_3/2_, the ratio of Ni^3+^/Ni^2+^ is enhanced, indicating higher p‐type conductivity [15, 31, 32]. More specifically, Ni^3+^ in NiO_X_ acts as an acceptor state by introducing holes into the valence band, thereby increasing p‐type conductivity and facilitating efficient hole transport. In contrast, Ni^2+^ does not contribute to hole creation. Therefore, a higher Ni^3+^/Ni^2+^ ratio directly correlates with improved hole extraction and transport properties in the HTL. The combined effects of dipole increment, work function enhancement, band offset reduction, and Ni^3+^/Ni^2+^ increment improve hole extraction and reduce interface recombination. Furthermore, the energy‐dispersive spectroscopy (EDS) mapping of Cl has been performed, showing that the majority of 4,5‐DI or 4,5‐D‐2‐MI molecules remain at the buried interface (Figure S10). Even with identical initial concentrations, 4,5‐D‐2‐MI shows a higher EDS Cl concentration (1 mg), especially closer to the NiO_X_ surface, likely due to enhanced interaction with Me‐4PACz. Additionally, a bandgap of 1.55 eV for perovskite films is observed, as confirmed by the UV–vis and Tauc plots of the perovskite films (Figure S11).

**FIGURE 3:**
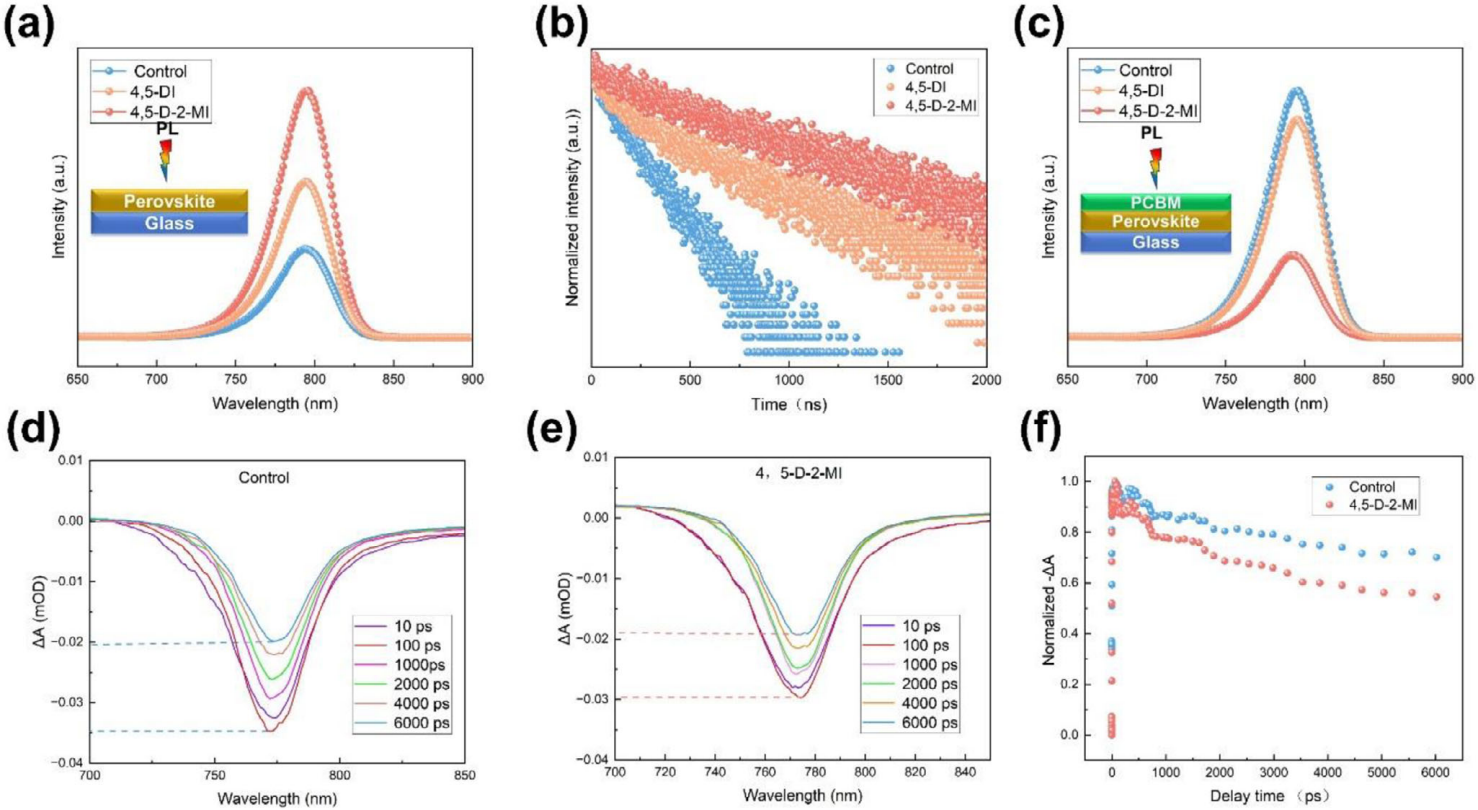
(a) PL and (b) TRPL analysis of control, 4,5‐DI‐ and 4,5‐D‐2‐MI‐based films (c) PL spectra of control, 4,5‐DI‐ and 4,5‐D‐2‐MI‐based films in the presence of PCBM. fs‐TAS spectra as a function of time delays, through 450 nm excitation within the wavelength region of 750–780 nm of (d) control and (e) 4,5‐D‐2‐MI‐based film. (f) Delta‐optical density (ΔA) of control and 4,5‐D‐2‐MI‐based film.

Photoluminescent (PL) analysis of the control and modified perovskite films on glass is conducted (see Figure 3a). As expected, the 4,5‐D‐2‐MI exhibited higher intensity, indicating strong radiative recombination. In addition, time‐resolved PL (TRPL) analysis indicates that the carrier lifetime (*τ*) of the modified films, especially the one with 4,5‐D‐2‐MI, is enhanced (see Figure 3b; Table S1). On the other hand, the reduced PL in glass/perovskite/PCBM, in the 4,5‐DI and 4,5‐D‐2‐MI‐based film arises from the rapid transfer of photo‐generated electrons from the perovskite to the PCBM layer, thereby suppressing radiative recombination within the perovskite (Figure 3c) [29]. Without a transport layer, the carriers survive for the longest time, indicating that the trap state capture centers are reduced, the probability of carrier capture is decreased, and also suggesting that non‐radiative recombination is inhibited. Further, we investigated the femtosecond transient absorption (fs‐TAS) to examine the impact of control and 4,5‐D‐2‐MI modification on the ultrafast carrier dynamics in the perovskite/PCBM films (see Figure 3d–f; Table S2). The ground‐state bleach (GSB) peak of the 4,5‐D‐2‐MI modified film decays faster. This is because the ground‐state electrons in the perovskite are excited to the excited state more quickly and participate in the charge transfer process. So, the consumption rate of the ground state electrons is accelerated, and the charge has a faster extraction rate from the VBM of the perovskite to the VBM of the hole transporting layer (HTL), resulting in the rapid decay of the GSB peak. This indicates that the 4,5‐D‐2‐MI‐modified film reduces the residence time of holes in the perovskite and the probability of hole‐electron recombination, thereby improving performance indicators such as the *V_OC_* and *FF* of the device.

**FIGURE 4:**
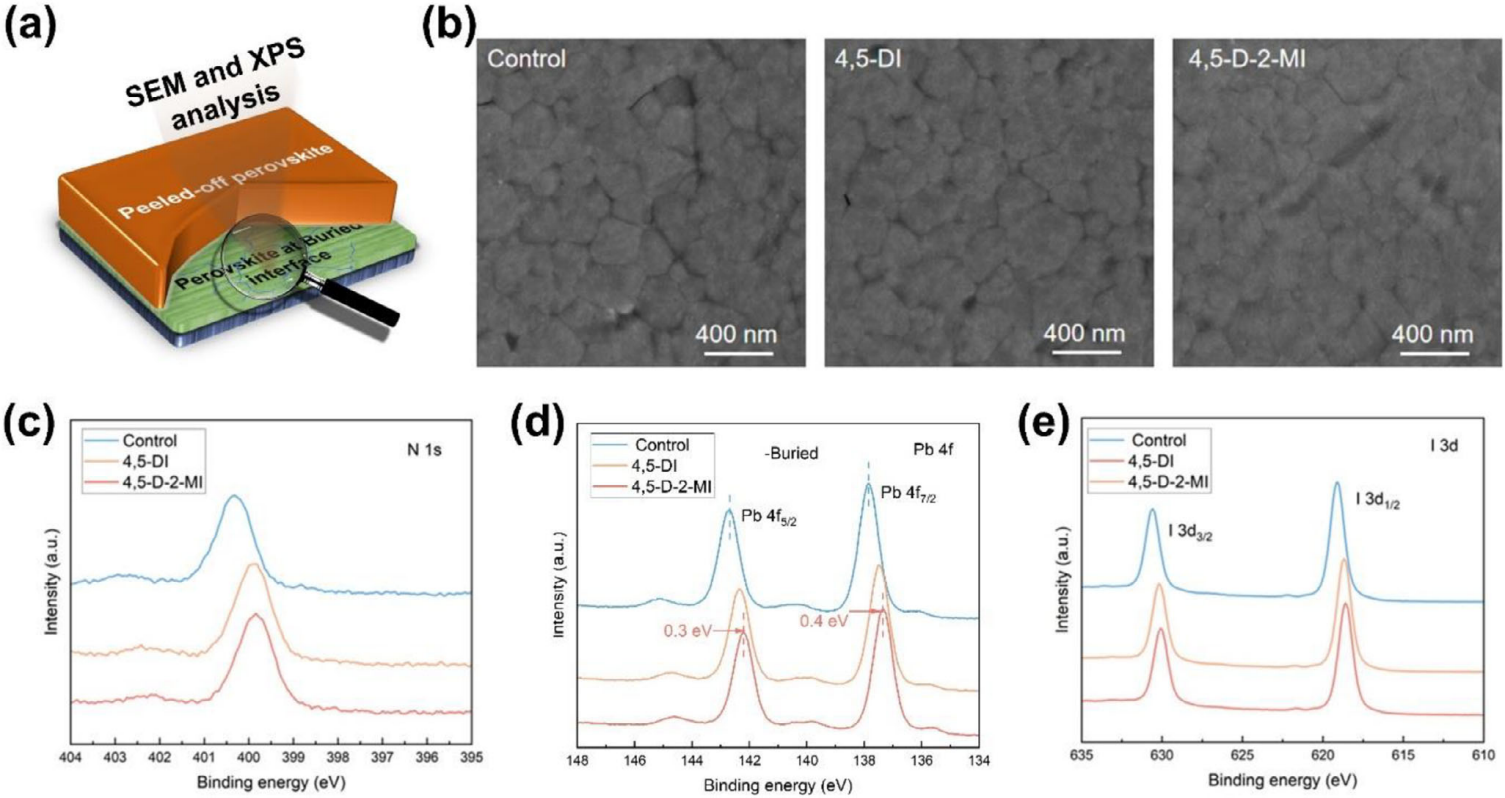
(a) Schematics of buried interface analysis, (b) SEM images of buried interface in the case of control, and the films modified with 4,5‐DI, and 4,5‐D‐2‐MI. (c) N 1s, (d) Pb 4f, (e) I 3d XPS profiles of buried interface in case of control, and the films modified with 4,5‐DI, and 4,5‐D‐2‐MI.

To further confirm the buried interface analysis, the upper perovskite was peeled off via our previously reported technique and conducted the SEM and XPS analysis as schematically presented in Figure 4a [33]. From the SEM analysis of the buried interface, 4,5‐D‐2‐MI exhibits adhesion in the grains as compared to the control or 4,5‐DI‐based films (Figure 3b). Interestingly, the control's N 1s peak intensity is lower and broader compared to the treated films, as the treatment introduces more nitrogen‐containing molecules (4,5‐DI or 4,5‐D‐2‐MI) at the buried interface. This scenario suggests stronger coordination or bonding of nitrogen to undercoordinated Pb sites or hydrogen bonding with iodide, which stabilizes the nitrogen's chemical environment. The observed shifts of the N 1s (−0.5 eV), Pb 4f (−0.3 eV), and I 3d peaks toward lower binding energies indicate that 4,5‐DI and 4,5‐D‐2‐MI molecules effectively interact with the perovskite (via Pb‐N interaction), increasing the local electron density around N, Pb, and I atoms; this chemical passivation stabilizes the interface by coordinating undercoordinated Pb^2+^ and reducing surface defects.

**FIGURE 5:**
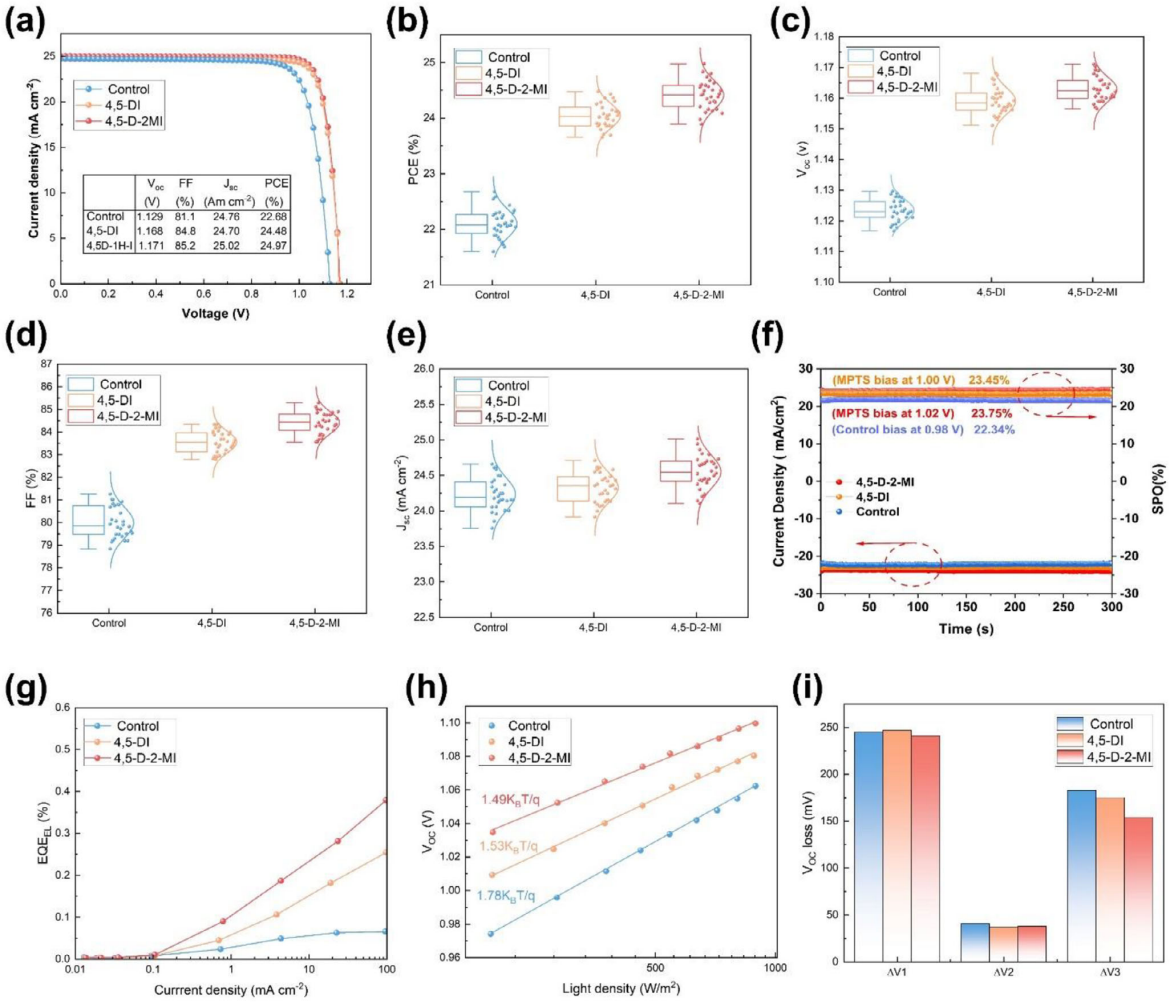
(a) Current density–voltage (*J*–*V*) curves and (b) statistical PCE, (c) *V_OC_*, (d) FF, and (e) *J_SC_* of control, 4,5‐DI‐ and 4,5‐D‐2‐MI‐based iPSCs. (f) Current density and steady‐state power output (SPO), measured for 300 s at a fixed voltage near the maximum power point (MPP) identified in the *J*–*V* curves. (g) *EQE_EL_* spectra, (h) *V_OC_*/light intensity curves, and (i) *V_OC_* loss analysis of control, 4,5‐DI‐ and 4,5‐D‐2‐MI‐based devices.

The complete process for preparing the materials and fabricating the device is provided in the Supporting Information. The device structure and energy level diagram of the target device are schematically presented in Figure S12, where the perovskite film formulation is *Cs_0.05_FA_0.85_MA_0.1_PbI_3_*. As confirmed from the reduced band offsets between the VBMs of HTL and perovskite, enhanced radiative recombination and reduced non‐radiative recombination by TAS, PL, and TRPL analysis, the *V_OC_* (from 1.129 to 1.171 V) and *FF* (81.1 to 85.2%) of the devices are impressively improved (Figure 5a). The target (4,5‐D‐2‐MI‐modified) devices exhibited an efficiency of 24.97%, with a *V_OC_* and *FF* of 1.171 V and 85.2%, respectively. The 4,5‐DI‐based device showed slightly lower performances than the target device, i.e., power conversion efficiency (PCE), *V_OC_*, and *FF* of 24.48%, 1.168 V, and 84.8%, respectively. On the other hand, the control device exhibits an efficiency of 22.68%, with *V_OC_* and *FF* values of 1.129 V and 81.1%, respectively. The external quantum efficiency (EQE) curves of the control and target device are provided in Figure S13. To further confirm the trend of improvement in photovoltaic parameters, we compared 30 devices from all three categories. The average values of PCE, FF, and *V_OC_* were improved (Figure 5b–d), accompanied by a slight increment in *J_SC_* (Figure 5e) following a similar trend to that of the champion devices from each group. Additionally, maximum power point (MPP) tracking has been performed to assess the stable steady‐state power output (SPO). As shown in Figure 5f, biases of 1.02, 1, and 0.98 V were applied to the control device, 4,5‐DI‐, and the 4,5‐D‐2‐MI‐based device, respectively. The control device produced a stable PCE of 22.35%, while the 4,5‐DI and 4,5‐D‐2‐MI devices produced the stable PCEs of 23.45% and 23.75%, respectively.

The enhanced photovoltage, PL analysis, and TAS observations all signal toward the low non‐radiative recombination. To solidify the proof, we conducted an in‐depth analysis of voltage loss for each category. From the electroluminescence (EL) spectroscopy measurements, the *EQE_EL_* of the modified devices is increased, especially for the 4,5‐D‐2‐MI‐based device (Figure 5g). A high *EQE_EL_* indicates that there are fewer recombination centers in the carrier transport process. In addition, from the relationship between *V_OC_* and light intensity shown in Figure 5h. It can be seen that the slope of 1.49K_B_T/q of the 4,5‐D‐2‐MI group is less than that of 1.78 K_B_T/q of the control group. The *V_OC_* loss analysis based on the Shockley–Queisser (S–Q) limit was conducted to evaluate the performance of the devices. According to the S–Q theory, the total open‐circuit voltage loss (*ΔV*) can be divided into three components: *qΔV = q(ΔV_1_+ΔV_2_+ΔV_3_)*, where *q* is the elementary charge, *ΔV_1_* is the thermodynamic loss, *ΔV_2_* represents radiative recombination loss, and *ΔV_3_* corresponds to non‐radiative recombination loss with ΔV_3_ calculated as *ΔV_3_ = ‐(kT/q)·ln(EQE_EL_)*. From which the *ΔV_3_* values are calculated as 187 mV for the control device, 176 mV for the 4,5‐DI‐modified device, and 159 mV for the 4,5‐D‐2‐MI‐modified device (Figure 5i). The complete voltage loss analysis is provided in the Supporting Information. The progressively reduced *ΔV_3_* demonstrates that imidazole‐based interface modifications effectively suppress non‐radiative recombination. This observation aligns well with the enhanced *EQE_EL_* and the reduced slope in the *V_OC–_* light intensity relationship, further confirming that imidazole derivatives improve device performance by reducing non‐radiative recombination losses, thereby increasing *V_OC_* and FF, and ultimately boosting the PCE of target devices.

At the same time, Nyquist plots were measured at a bias voltage of 0.99 V in a dark environment (Figure S14 and Table S3). Compared to the control, the devices treated with imidazole exhibit an increase in recombination resistance (*R_rec_*) and a decrease in series resistance (*R_S_*). A single semicircle in the Nyquist plots represents *R_rec_*. Among them, 4,5‐D‐2‐MI still shows the best performance. This phenomenon indicates that the defect state density at the buried interface is reduced, which is beneficial for carrier transport and effectively prevents charge recombination, thereby improving the *V_OC_* and FF of the device. From the space charge limited current (SCLC) analysis, as shown in Figure S15, the well‐filling limit voltage (*V_TFL_*) in the target device decreased. The *V_TFL_* of the 4,5‐D‐2‐MI‐based device is the smallest, decreasing from *V_TFL_*_ = _0.59 V of the Control group to *V_TFL_*_ = _0.51 V of the 4,5‐D‐2‐MI group. According to the trap density formula (*n_t_ = 2εε_0_VTFL/eL^2^*), the hole trap density is reduced from 5.25×10^15^ to 4.54×10^15^ cm^−^^3^ upon the post‐treatment of Me‐4APCz via 4,5‐D‐2‐MI. Similarly, the reduced dark current observed in the modified devices, which can be attributed to enhanced grain size and fewer grain boundaries (Figure S16) [34].

**FIGURE 6:**
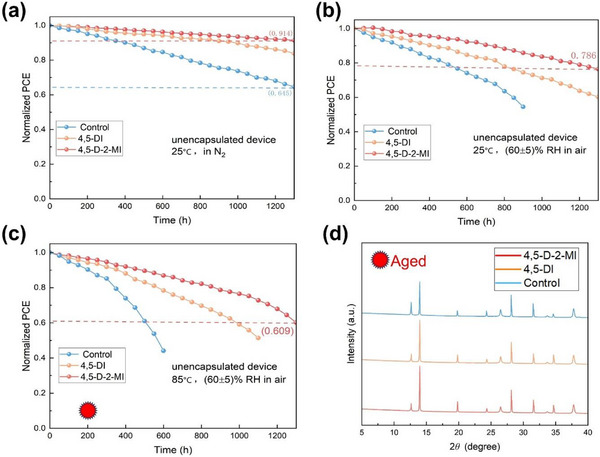
Stability comparison of unencapsulated iPSCs stored (a) in an N_2_ atmosphere at room temperature, (b) in RH = 60±5%, room temperature, and air, (c) in RH = 60±5%, at 85°C. (d) XRD of aged films (200 h in RH = 60±5%, at 85°C) for each case.

Ultimately, we conducted three separate studies to investigate the long‐term stability of unencapsulated devices under various storage conditions. In a N_2_ environment at 25°C (Figure 6a), the best‐performing 4,5‐D‐2‐MI perovskite device retained 91.4% of its original efficiency after 1300 h, while the control device maintained only 64.5%. In addition, stability tests were conducted under two further storage conditions: 25°C, 60±5% relative humidity (RH) in air and 85°C, 60±5% RH in air (Figure 6b). The 4,5‐D‐2‐MI device maintained 78.6% of the original efficiency after 1300 h, while the control device only had half of the original efficiency after 900 h. Under similar conditions, but at 85°C (Figure 6c), the 4,5‐D‐2‐MI device still maintained 60.9% of its original efficiency after 1300 h, whereas the Control device only retained 40% of its original efficiency after 600 h. We can attribute this enhanced stability toward the enhancement in the quality of the perovskite film deposited on the post‐treated Me‐4PACz and improvement in the buried interface of target devices. Under similar conditions, unpackaged perovskite films were stored from each category for 200 h, and XRD analysis was conducted on these aged films (Figure 6d). The attenuation of the perovskite in the (100) direction and the appearance of the PbI_2_ peak of the control film were more obvious. Nevertheless, the modified films, particularly the one based on 4,5‐D‐2‐MI, exhibited the lowest PbI_2_ peaks, indicating that the perovskite distortion is minimum in the target films.

## 3 Conclusions

A simple, yet effective, post‐treatment strategy has been demonstrated to overcome the intrinsic limitations of Me‐4PACz at the buried interface of inverted perovskite solar cells. By introducing chlorinated imidazole derivatives (4,5‐DI and 4,5‐D‐2‐MI) at the Me‐4PACz surface converted the surface wettability is enhanced, enabling improved perovskite film coverage and quality. Furthermore, the buried interfacial properties are enhanced as the strengthened carbazole–imidazole interactions and additional interfacial dipoles tune the energy‐level alignment, reduce interfacial defects, and facilitate more efficient charge extraction. Consequently, the 4,5‐D‐2‐MI‐treated devices achieved a champion efficiency of 24.97%, while 4,5‐DI delivered 24.48%. Both exhibited superior long‐term stability under high humidity and high temperature conditions as compared to untreated counterparts. This work highlights the potential of post‐treatment molecular engineering as a facile strategy to enhance the interfacial properties of SAM/perovskite interfaces and improve the performance and stability of iPSCs.

## Funding

Natural Science Foundation of Top Talent of SZTU (grant no. GDRC202422), Shenzhen Science and Technology Program (no. 20231128110928003), Human Resources and Social Security Administration of Shenzhen Municipality.

## Conflicts of Interest

The authors declare no conflicts of interest.

## Supporting information




**Supporting file**: advs73565‐sup‐0001‐SuppMat.docx.

## Data Availability

The data that support the findings of this study are available from the corresponding author upon reasonable request.
