# Supplementary material for: Imidazole Post‐Treated Self‐Assembled Monolayers for Inverted Perovskite Solar Cells
Source: Adv Sci (Weinh). 2025 Dec 26;13(14):e18676. doi: 10.1002/advs.202518676 (PMC12970168; doi:10.1002/advs.202518676)
Supplement: Supplementary file 1 — Supporting file: advs73565‐sup‐0001‐SuppMat.docx. [file ADVS-13-e18676-s001.docx]

**Supporting Information**

**Imidazole Post-Treated Self-Assembled Monolayers for Inverted Perovskite Solar Cells**

Ihtesham Ghani, ^a,b,c,^ Shi Tingshu,^a,c,^ Shehzad Ahmed,^d,^ Zhuo Hongbin ^a,b^*, Zhu Zirun,^a,c^ Yu Zhang,^c^ Peng You, ^c^Imran Muhammad,^e^ Tang Zeguo,^c,^* Danish Khan,^c,^*

**^a^** College of Applied Technology, College of Physics and Optoelectronic Engineering, Shenzhen University, Shenzhen 518060, China

**^b^** Shenzhen Key Laboratory of Ultraintense Laser and Advanced Material Technology, Center for Advanced Material Diagnostic Technology, and College of Engineering Physics,

Shenzhen Technology University, Shenzhen 518118, China

**^c^** The College of New Materials and New Energies, Shenzhen Technology University, Lantian Road 3002, Pingshan, Shenzhen 518118, China.

^d^ China-UK Low Carbon College, Shanghai Jiao Tong University, Lingang, Shanghai 201306, China

^e^ National Industry-Education Platform of Energy Storage, Tianjin University, 135 Yaguan Road, Tianjin, 300350, P.R. China.

E-mail: [zhuohongbin@sztu.edu.cn](mailto:zhuohongbin@sztu.edu.cn), [tangzeguo@sztu.edu.cn](mailto:tangzeguo@sztu.edu.cn), [khandanish@sztu.edu.cn](mailto:khandanish@sztu.edu.cn)

**Computational Details**

Density functional theory (DFT) calculations were performed using the Vienna Ab initio Simulation Package (VASP) with the projector-augmented wave (PAW) method^[1]^. The exchange–correlation potential was described by the generalized gradient approximation (GGA) in the Perdew–Burke–Ernzerhof (PBE) formulation, which is widely adopted for surface modeling^[2]^. Long-range van der Waals interactions were included via the DFT-D3 correction of Grimme, ensuring accurate treatment of weak physisorption and dispersion effects^[3]^. A kinetic energy cutoff of 550 *eV* was employed to expand the plane-wave basis, and Brillouin zone integrations were performed with Monkhorst–Pack k-point grids of 3×5×1 for surface models^[4]^. Geometry optimizations were carried out until the total energy converged within 10^-5^ *eV* and atomic forces were below 0.001 *eV Å^-1^*. Surfaces were represented by slab models, typically consisting of four atomic layers, with the bottom two layers fixed to mimic the bulk structure and the upper layers allowed to relax. A vacuum spacing of at least 25 *Å* was applied along the z-direction to avoid interactions between periodic images. Adsorption energies were determined as:$E_{form}=E_{NiOx+ads}-E_{NiOx}-\mu_{ads}$where each term corresponds to the optimized energy of the combined system, the clean slab, and the isolated molecule. Gas-phase species were modeled in a cubic box with a vacuum separation of at least 25 *Å* and sampled at the Γ point. To investigate charge redistribution, charge density difference (*CDD*) maps were generated defined as:$\Delta\rho=\rho_{NiOx+ads}-\rho_{NiOx}-\rho_{NiOads}$where *ρ* represents the charge density of the combined and isolated systems. Complementary molecular-level calculations were performed using Gaussian 16 to evaluate the electrostatic potential (*ESP*) distribution and dipole moments of isolated adsorbate molecules^[5]^. Geometry optimizations and frequency calculations were carried out using the B3LYP functional with the 6-311++G(d,p) basis set, which balances computational cost with accuracy for molecular properties^[6]^. The *ESP* maps were generated from the converged wave functions, providing insights into regions of electron-rich and electron-deficient character that dictate adsorption orientation and reactivity. Dipole moments were extracted directly from the electronic wavefunction analysis and were used to assess the polarity of adsorbates and their potential influence on adsorption configurations and reaction kinetics at the catalyst interface^[7]^.

### Experimental Section

### Materials: Phenethylammonium chloride (PEACl, 99.5%), (4- (3,6-dimethyl-9H-carbazole-9-yl) butyl) phosphonic acid(Me-4pacz,99.5%), Formamidinium iodide (FAI, 99.9%), methylammonium iodide (MAI, 99.5%), methylammonium Chloride (MACl,99.9%) Cesium iodide (CsI, 99.9%), Lead iodide (PbI2, 99.999%), Fullerene (C60, 99.9%), and 2,9-dimethyl-4,7- diphenyl-1,10-phenanthroline (BCP, 98%) were purchased from Xi’an Polymer Light Technology. NiOX nanoparticles were procured from Advanced Election Technology, Chlorobenzene (CB,anhydrous,99.8%), N,N-dimethylformamide (DMF, anhydrous, 99.8%), isopropyl alcohol (IPA), and dimethyl sulfoxide (DMSO, anhydrous, ≥99.9%) were purchased from Sigma-Aldrich.4,5-Dichloroimidazole, 4,5-Dichloro-2-methylimidazole were purchased from Shanghaiyuanye Bio-Technology Co., Ltd.

### Device Fabrication: The FTO glass (2.45 *mm*×2.45 *mm*, 7-8 *Ω sq⁻¹)* was ultrasonically cleaned in water with alkaline glass cleaner, deionized water, isopropanol, and ethanol for 20 *minutes* each. It was then dried with a nitrogen gun and treated with ultraviolet ozone for 15 *minutes*. The NiO_X_ dispersion (10 *mg ml⁻¹* in deionized water) was sonicated in water for 10 *minutes* and filtered through a 0.22-*μm* PES membrane before use. 80 *μl* of this dispersion was spin-coated onto the FTO at 2000 *rpm* for 30 *s* and then annealed at 150 *°C* for 30 *minutes*. Immediately after that, it was transferred into a nitrogen-filled glove box. 100 *μl* of the Me-4PACz solution (0.5 *mg ml⁻¹* in ethanol) was spin-coated at 4000 *rpm* for 30 *s* and annealed at 100 *°C* for 10 *minutes*. Then, 80 *μl* of the 4,5-DI or 4,5-D-2-MI solution (0.5 *mg ml⁻¹*) was spin-coated at 4000 *rpm* for 30 *s* and annealed at 70 *°C* for 10 *minutes*. For the 1.5 M *Cs_0.05_FA_0.85_MA_0.1_PbI_3_* perovskite solution, it was dissolved in 1 *ml* of a DMF: DMSO mixed solvent ( v/v=4:1). After stirring at 60 *°C* for 2 *hours*, PbI_2_ and MACl were added with a molar ratio of 10% and 12%, respectively. The perovskite solution was then spin-coated at 1000 *rpm* for 10 *s* and then at 4000 *rpm* for 40 *s*. At 45 *s* (5 *s* before the end of spin-coating), 160 *μl* of chlorobenzene (CB) antisolvent was rapidly and steadily dropped. Subsequently, the film was annealed in a nitrogen-filled glove box at 100 *°C* for 30 *minutes*. PEACl was dissolved in IPA and spin-coated at 4000 *rpm* for 30 *s* and then annealed at 70 *°C* for 5 *minutes*. Then, 35 *nm* thick C60 and 6 *nm* thick BCP were successively evaporated under a vacuum of 1×10⁻⁵ *Pa*, and 100 *nm* thick Ag was evaporated under a vacuum below 1×10⁻¹⁴ *Pa*.

**Film characterization:** Scanning electron microscopy (SEM), energy-dispersive spectroscopy (EDS) images of perovskite films were obtained using Gemini SEM 300. Atomic Force Microscope (AFM) and Kelvin Probe Force Microscopy (KPFM) were conducted on Oxford Instruments Asylum Research, Cypher S. The samples have been used to measure the UPS spectrum under ESCALAB 250Xi (Thermo Scientific) and HE Izon Photon Source (21.22 *eV*) under ultra-high vacuum (<10^-7^ *Pa*) and a partial pressure of -10 *V* were applied between the samples and detectors. Use a clean gold film to combine the ultraviolet photoelectron spectroscopy (*UPS*) spectrometer to calibrate the energy marking, X-ray photoelectron spectroscopy (*XPS*) measurements were performed using a ThermoVG Scientific ESCALAB 250 X-ray photoelectron spectrometer with a monochromatic Al (Kα) (1,486.6 *eV*) X-ray source Thermo Fisher Scientific (Nicolet IS50) and Fluotime 300, Picoquant was applied to measure Steady-state photoluminescence (*PL*) spectra and time-resolved photoluminescence (*TRPL*) spectra. X-ray diffraction (*XRD*) is completed by Rigaku Smartlab diffraction meter equipped with CU K-α radiation source (λ = 1.5405å). The femtosecond transient absorption spectroscopy (fs-TAS) was performed using a 1 kHz, 85 *fs* Ti: sapphire ultrafast laser amplifier with the wavelength of 450 *nm* having the pump-probe transient absorption spectrometer (Helios, Ultrafast System).

**Device characterization:** Using the solar simulator to connect to Keithley 2400 digital sources, it records the current density-voltage (*JV*) curve of perovskite solar cells under AM1.5G radiation, and calculates the power of the valid light area of 0.06 *cm-2* batteries. Power conversion efficiency (*PCE*), current density and steady-state power output (*SPO*), and measures its space charge limited current (*SCLC*) in the dark environment. External quantum efficiency (*EQE*) is obtained by monochrome lighting QE-R3011 (Enli Tech), Oriel CornerStone260 1/4 m monochrome, and ENLITECH's Oriel 70613 *ns* QTH light, and within 300-900 *nm*, it is obtained. Electrochemical impedance spectrum (*EIS*) measurement is performed at Par Ametek, Versastat 3. For stability testing, the device under test is subjected to the corresponding environmental conditions and taken out regularly for measurement. After each measurement, they will return to their respective storage environments for subsequent measurements.

**Photovoltage loss (*V_OC_*, loss) *EL* and *EQE_EL_*** were performed by applying external voltage/current sources through the devices (REPS, Enlitech). The detailed *V*_OC_, loss can be described by the equation listed below:

qΔV $=$ E_g_ $-$ qV_OC_

$=$ $($E_g_ $-$ q$V_{OC}^{SQ}) +$ $($q$V_{OC}^{SQ}-$ q$V_{oc}^{rad}) +$ $($q$V_{oc}^{rad}-$ qV_OC_$)$

$=$ $($E_g_ $-$ q$V_{OC}^{SQ}+$ q$\Delta V_{OC}^{SQ}) +$ q$\Delta V_{oc}^{rad}+$ q$\Delta V_{oc}^{non-rad}$

$=$ q$($ΔV_1_$+$ΔV_2_$+$ΔV_3_$)$ (1)

Where *q*, Δ*V*, *E_g_* are the elementary charge, the total voltage loss, and the bandgap of perovskite, respectively. $V_{OC}^{SQ}$is the Shockley-Queisser limit of open circuit voltage, $V_{oc}^{rad}$ is the *V*_OC_ without non-radiative recombination occurring in PSCs. $\Delta V_{OC}^{SQ}$ is the *V*_OC_ loss due to the non-ideal *EQE* above the bandgap, ∆$V_{oc}^{rad}$ is the *V*_OC_ loss due to the sub-bandgap radiative recombination, and *∆* $V_{oc}^{non-rad}$ is the *V*_OC_ loss of non-radiative recombination. As a consequence, the energy loss can be divided into three parts, Δ*V*_1_, Δ*V*_2,_ and Δ*V*_3_ representing radiative recombination above *E*_g_, energy loss from blackbody radiation and voltage loss induced by the nonradiative recombination, respectively.

The *V*_OC_ of a solar cell can be calculated by the equation: ^4^

V_OC_$= \frac{k_{B}T}{q}\ln\left( \frac{J_{sc}}{J_{0}} \right)$ (2)

where *q*, *k_B_*, *T*, *J*_SC_, *and J_0_* represents the element charge, Boltzmann constant, temperature, short-circuit current, and dark saturation current, respectively. The *J*_SC_ and *J*_0_ can be described as:

J_SC_$=$q$\int_{0}^{\infty} {EQE}_{PV}(E)$Ф_AM1.5_$($E$)dE$ (3)

J_0_$=\frac{q}{{EQE}_{EL}}\int_{0}^{\infty} {EQE}_{PV}(E)$Ф_BB_$($E$)dE$ (4)

Ф_BB_$($E$)$ $=\frac{{2\pi E}^{2}}{h^{3}c^{2}}\frac{1}{\exp\left( \frac{E}{k_{B}T} \right)-1}$ (5)

where *EQE*_PV_ and *EQE*_EL_ are photovoltaic external quantum efficiency and electroluminescence external quantum efficiency, respectively. whereas, *Ф*_AM1.5_ and *Ф*_BB_ are the solar cell radiative spectrum and black-body radiative spectrum, respectively and *c* is the light speed in a vacuum.

According to the Schokley-Queisser limit (S-Q limit): (1) The *EQE*_PV_ is described with the Heaviside step function, where *EQE*_PV_$($*E*$)$ $=\left\{ \begin{aligned} 1, E\geq E_{g} \\ 0, E＜E_{g} \end{aligned} \right.$; (2) only the photons with energy larger than bandgap (*E*_g_) are absorbed; (3) all recombination is radiative (*EQE*_EL_*＝*1).

Therefore, *J*_SC_ and *J*_0_ in S-Q limit can be written as:

$J_{SC}^{SQ}=$ q$\int_{E_{g}}^{\infty} Ф_{AM1.5}$(E)$dE$ (6)

$J_{0}^{SQ}=$ q$\int_{E_{g}}^{\infty} Ф_{BB}$(E)$dE$ (7)

Therefore, *V*_OC_ S-Q limit is:

$V_{OC}^{SQ} = \frac{k_{B}T}{q}\ln\left( \frac{J_{SC}^{SQ}}{J_{0}^{SQ}} \right)$ (8)

Considering the theory of the S-Q limit, $V_{OC}^{SQ}$ can be degraded to *V*_oc_ with three components of loss. The first *Voc* loss component is due to the non-ideal *EQE_PV_*, which is less than 100％. In this situation, short-circuit current can be expressed as:

J_sc_ $=$ q$\int_{0}^{\infty} {EQE}_{PV}(E)$Ф_AM1.5_(E)$dE$ (9)

The $\Delta V_{OC}^{SQ}$ was calculated as below:

$\Delta V_{OC}^{SQ}$ $=$ $V_{OC}^{SQ}- \frac{k_{B}T}{q}\ln\left( \frac{J_{SC}}{J_{0}^{SQ}} \right)=\frac{k_{B}T}{q}\ln\left( \frac{J_{SC}^{SQ}}{J_{SC}} \right)$ (10)

The second *V*_OC_ loss component originates from the energy loss related to the extra thermal radiation of solar cells in the dark. The *EQE*_PV_ extends into the sub-bandgap region where the black-body radiation increases on the reduction in photo energy. Thus, this sub-bandgap *EQE*_PV_ increases the dark saturation current. The short-circuit current $J_{\mathrm{sc}}^{rad}$ is equal to *J*_SC_, and dark saturation current in this condition can be written as:

$J_{0}^{rad}=$ $q\int_{0}^{\infty} {EQE}_{PV}(E)$Ф_BB_(E)$dE$ (11)

therefore, the radiative *V*_OC_ loss, $\Delta V_{oc}^{rad},\mathrm{is}$:

$\Delta V_{\mathrm{oc}}^{rad}$ $=$ $\frac{k_{B}T}{q}\ln\left( \frac{J_{SC}}{J_{0}^{SQ}} \right)-$ $\frac{k_{B}T}{q}\ln\left( \frac{J_{sc}}{J_{0}^{rad}} \right)=\frac{k_{B}T}{q}\ln\left( \frac{J_{0}^{rad}}{J_{0}^{SQ}} \right)$ (12)

The third *V*_OC_ loss component, $\Delta V_{oc}^{nonrad}$, which is attributed to the non-radiative recombination in the device, can be calculated as:

$\Delta V_{oc}^{nonrad}$ $=$ $\frac{k_{B}T}{q}\ln\left( \frac{J_{sc}}{J_{0}^{rad}} \right)$ $-$ V_OC_ (13)

According to Equation S8 and S15, $J_{0}^{rad}=$ *EQE_EL_*·*J*_0_:

$\Delta V_{oc}^{nonrad}$ $= \frac{k_{B}T}{q}\ln\left( \frac{J_{SC}}{{EQE}_{EL}\cdot J_{0}} \right)$ $-$ $\frac{k_{B}T}{q}\ln\left( \frac{J_{SC}}{J_{0}} \right)$

$= -\frac{k_{B}T}{q}\ln\left( {EQE}_{EL} \right)$ (14)

**Supporting Figures**

**
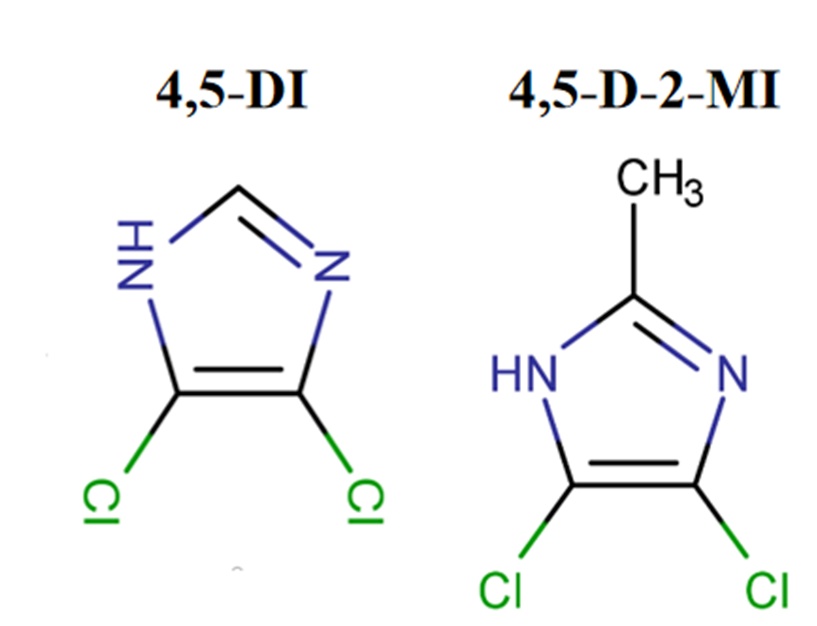
**

**Figure S1.** Chemical structures of 4,5-DI and 4,5-D-2-MI.

**
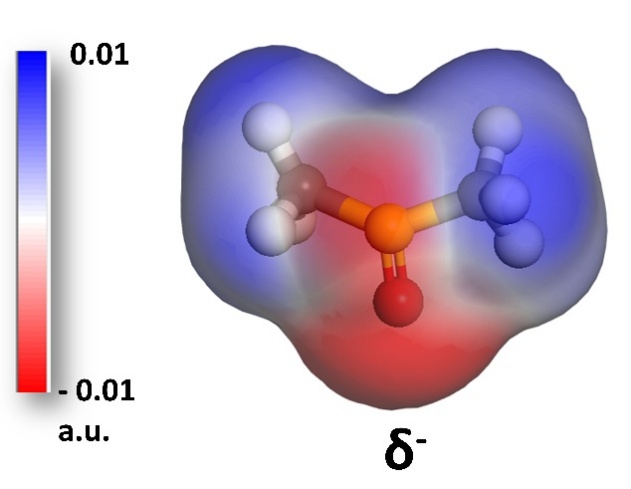
**

**Figure S2.** ESP maps of DMSO (perovskite’s precursor solvent).


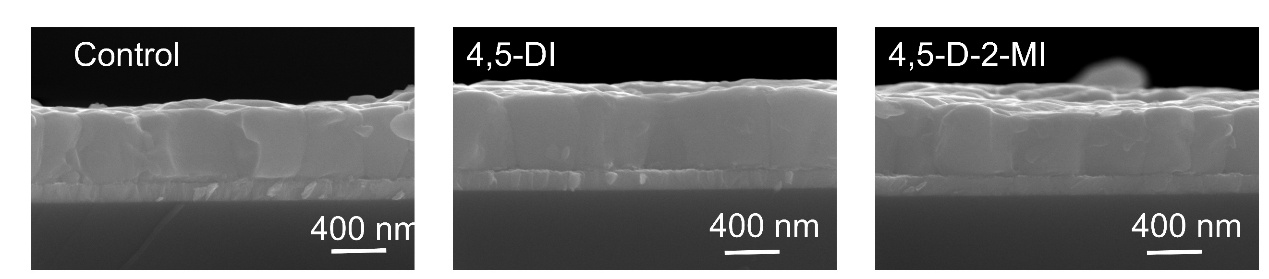


**Figure S3.** Cross-sectional SEM images of control, 4,5-DI-, and 4,5-D-2-MI- based films.


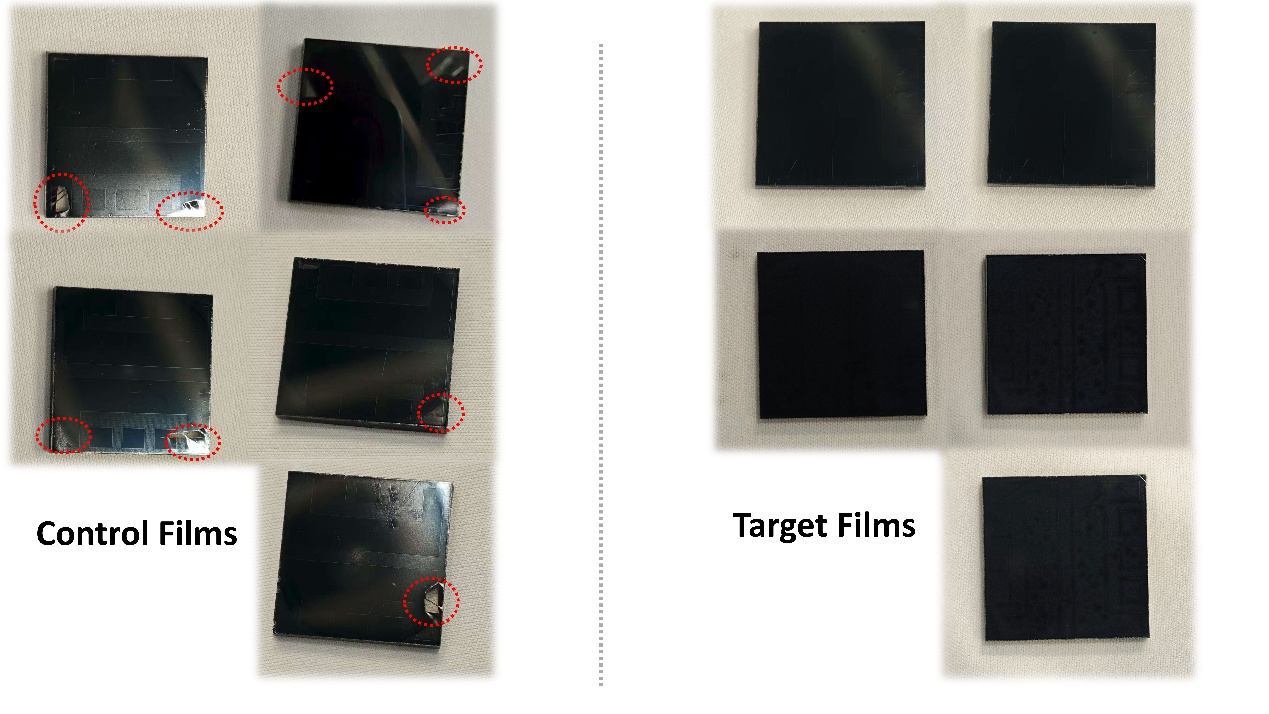


**Figure S4.** Photographs of Control, and 4,5-D-2-MI-based films.


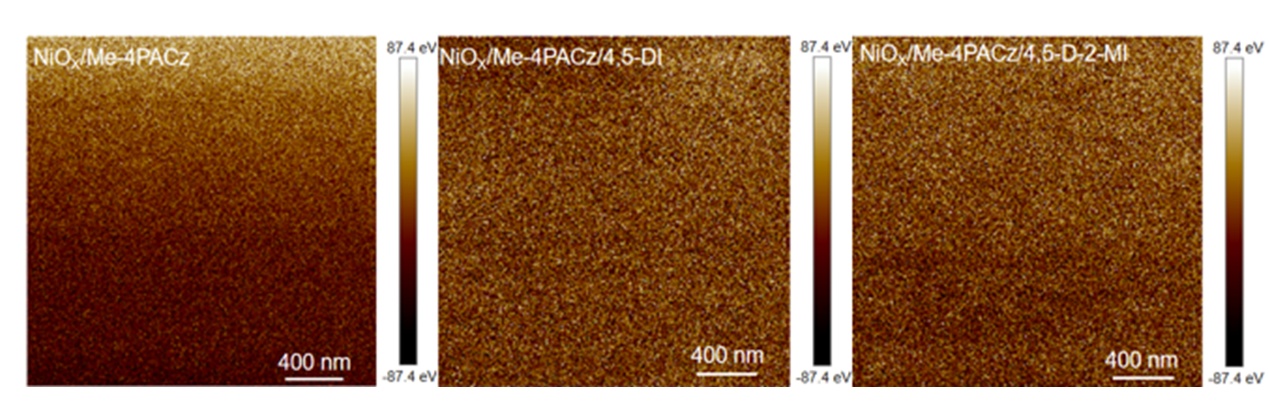


**Figure S5.** KPFM images of NiO_X_/Me-4PACz, NiO_X_/Me-4PACz/4,5-DI-, and NiO_X_/Me-4PACz/4,5-D-2-MI films.


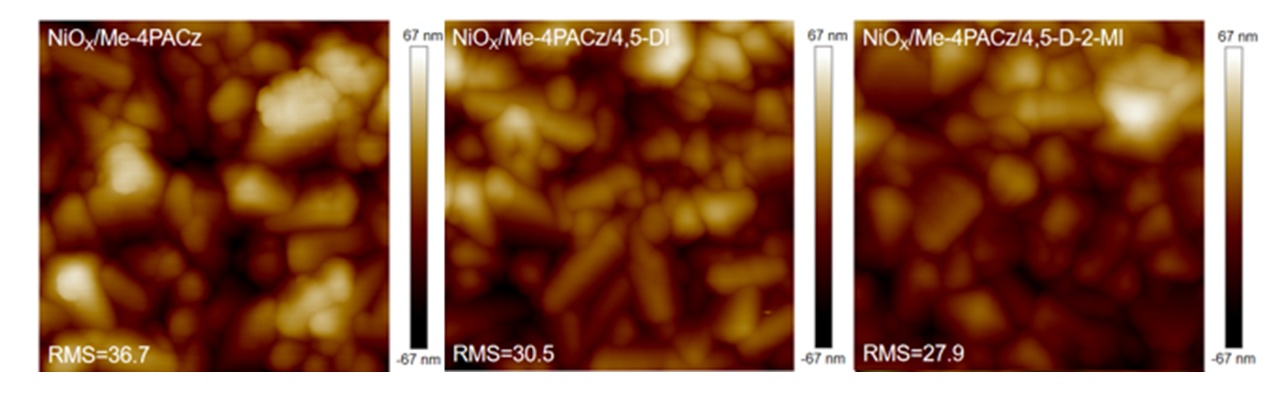


**Figure S6.** AFM images of NiO_X_/Me-4PACz, NiO_X_/Me-4PACz/4,5-DI, and NiO_X_/Me-4PACz/4,5-D-2-MI.


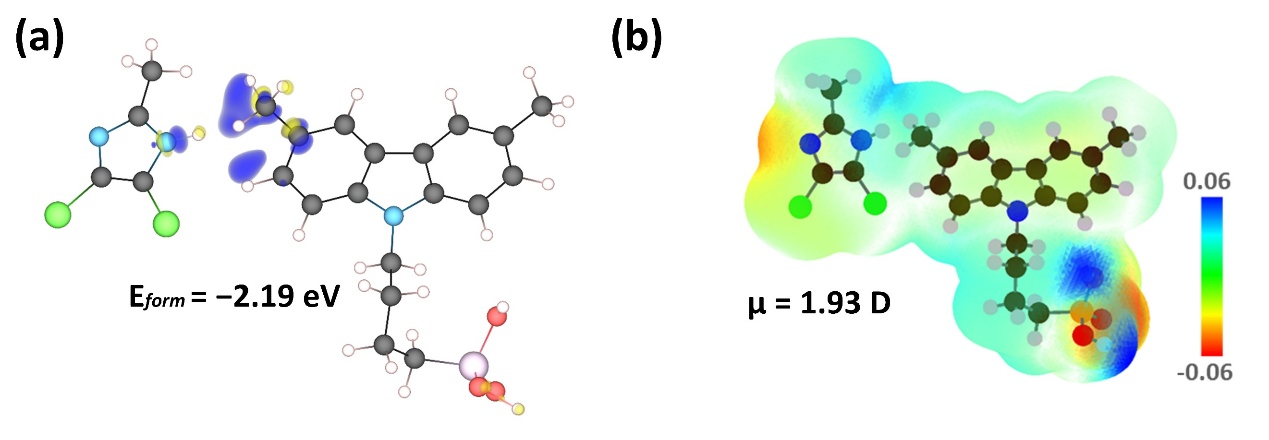


**Figure S7**. Formation energy (*E_form_*) between the Me-4PACz and 4,5-D-2-MI, and charge transfer between the methyl of Me-4PACz and -NH of 4,5-D-2-MI (b) ESP and dipole moment (*µ*) of Me-4PACz:4,5-D-2-MI.


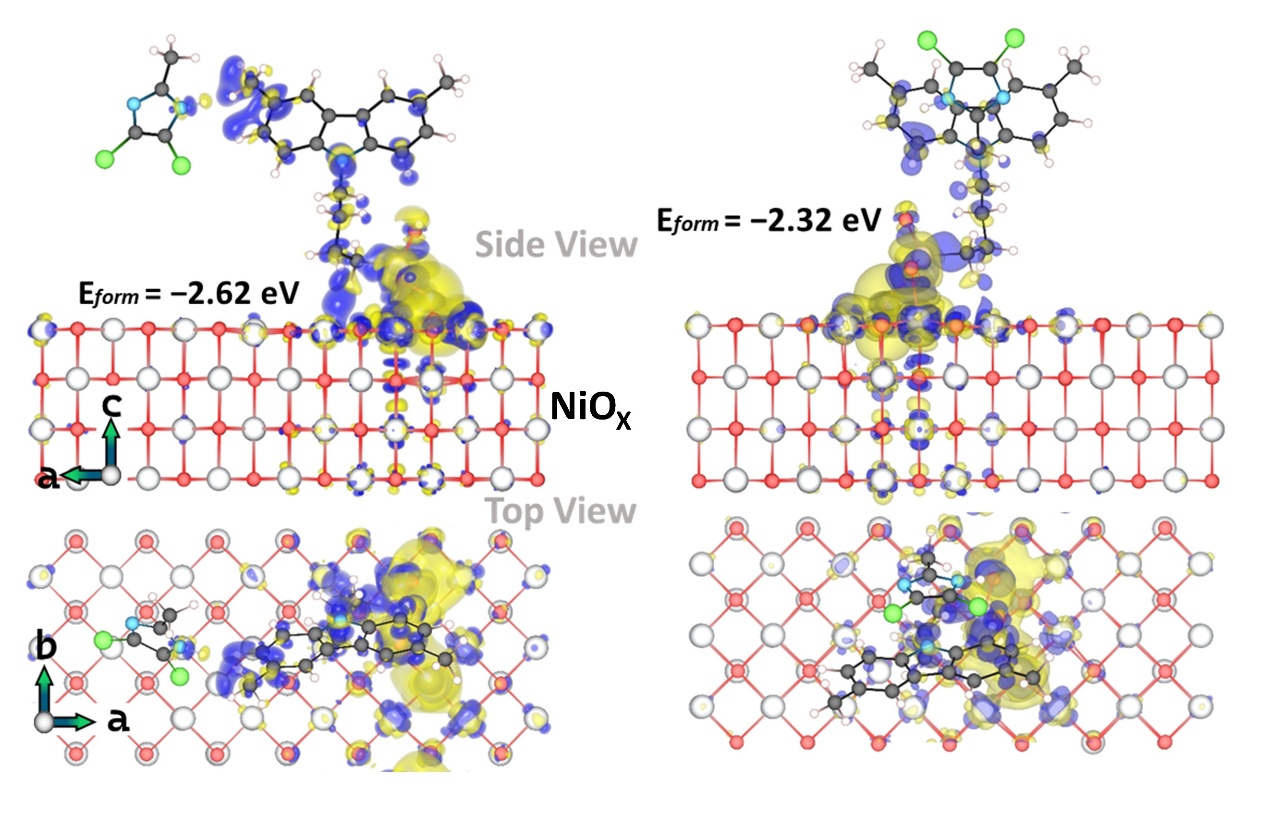


**Figure S8.** Side view and top view of two types of interaction (hydrogen and π-π), charge transfers and formation energies of NiOX and Me-4PACz in each case.

**
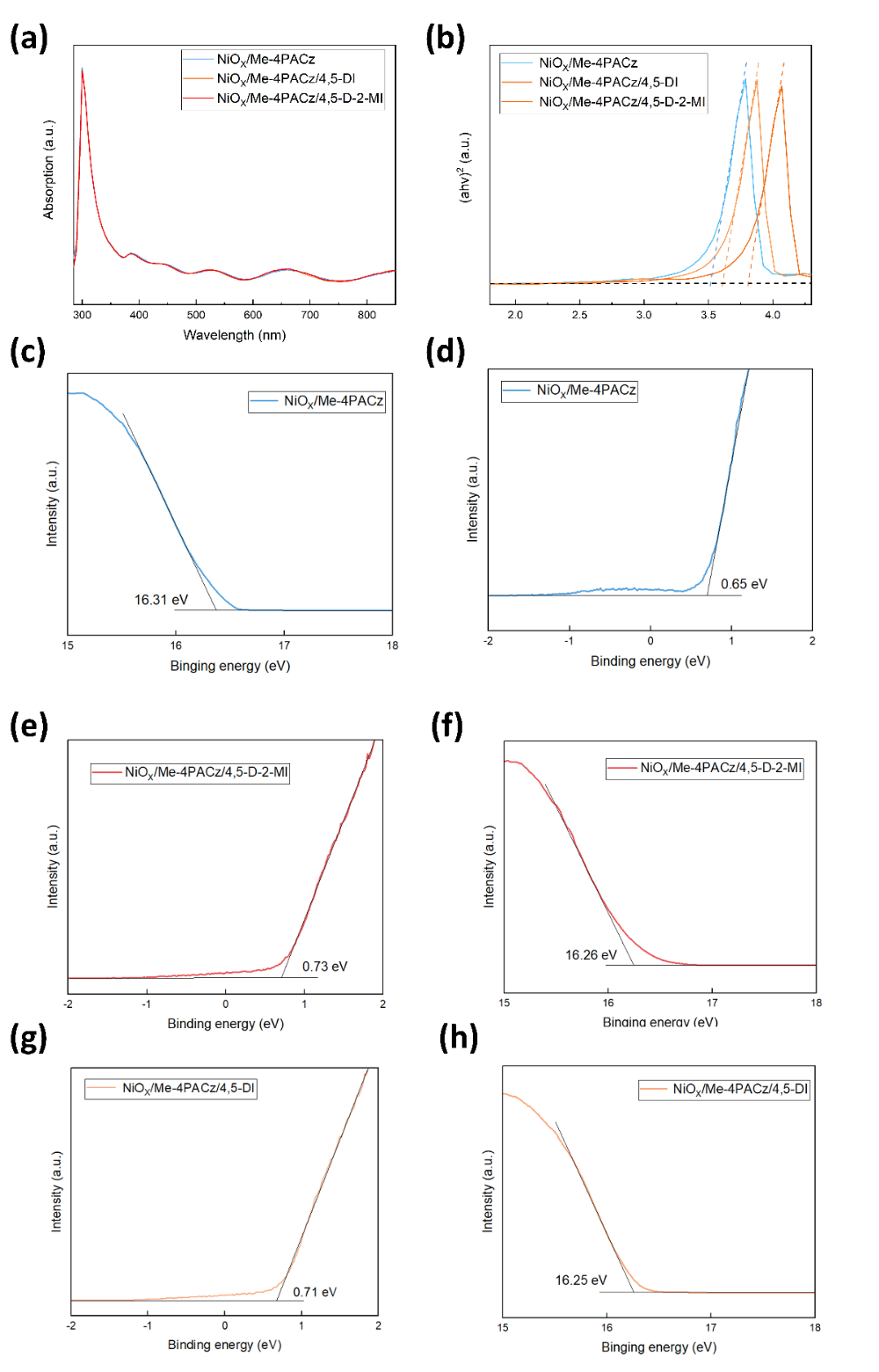
Figure S9.** (a) UV-vis absorption spectra and (b) extracted Tauc plots of NiO_X_/Me-

4PACz, NiO_X_/Me-4PACz/4,5-DI, and NiO_X_/Me-4PACz/4,5-D-2-MI. (c, e, g) Fermi edges and (d, f, h) secondary electron cutoff (SECO) of NiO_X_/Me-4PACz.

**
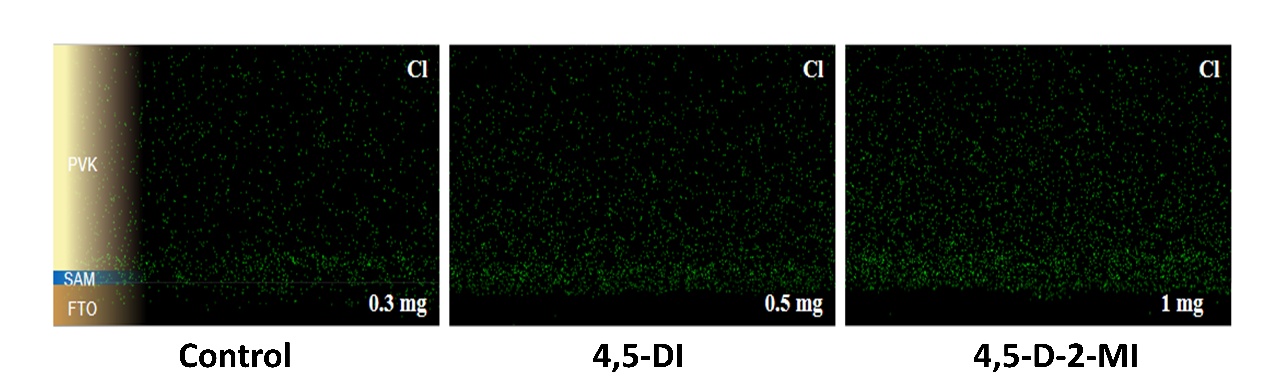
**

**Figure S10.** EDS mapping/concentration of Cl atoms in control, 4,5-DI-, and 4,5-D-2-MI-based films.

**
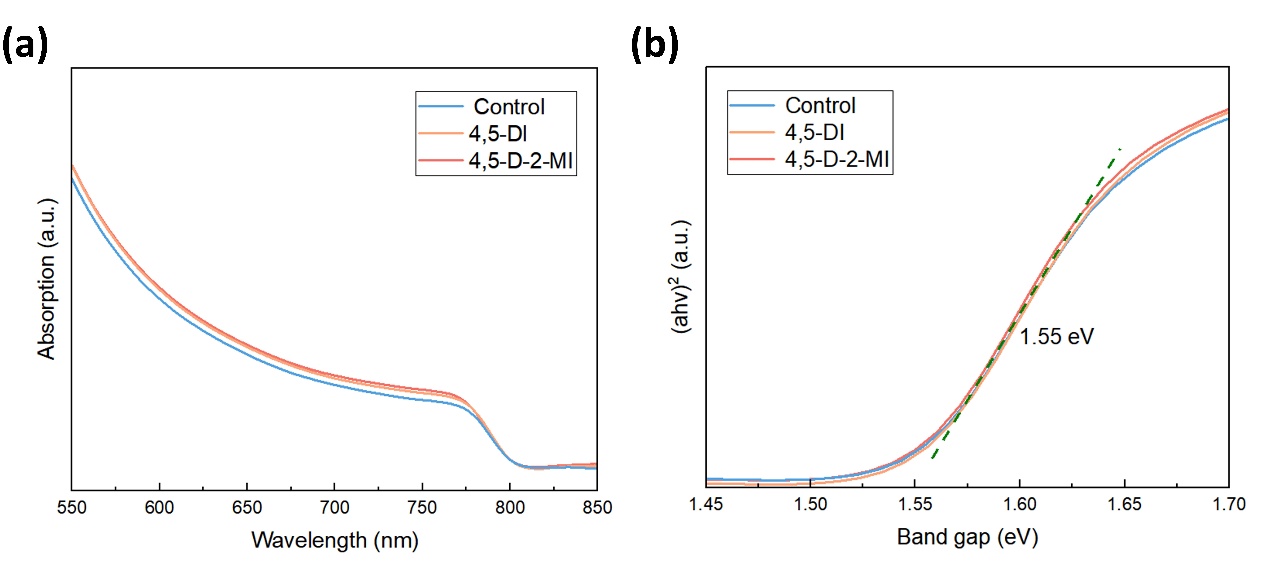
**

**Figure S11.** UV-vis absorption and tauc plot of control, 4,5-DI-, and 4,5-D-2-MI-based films.

**
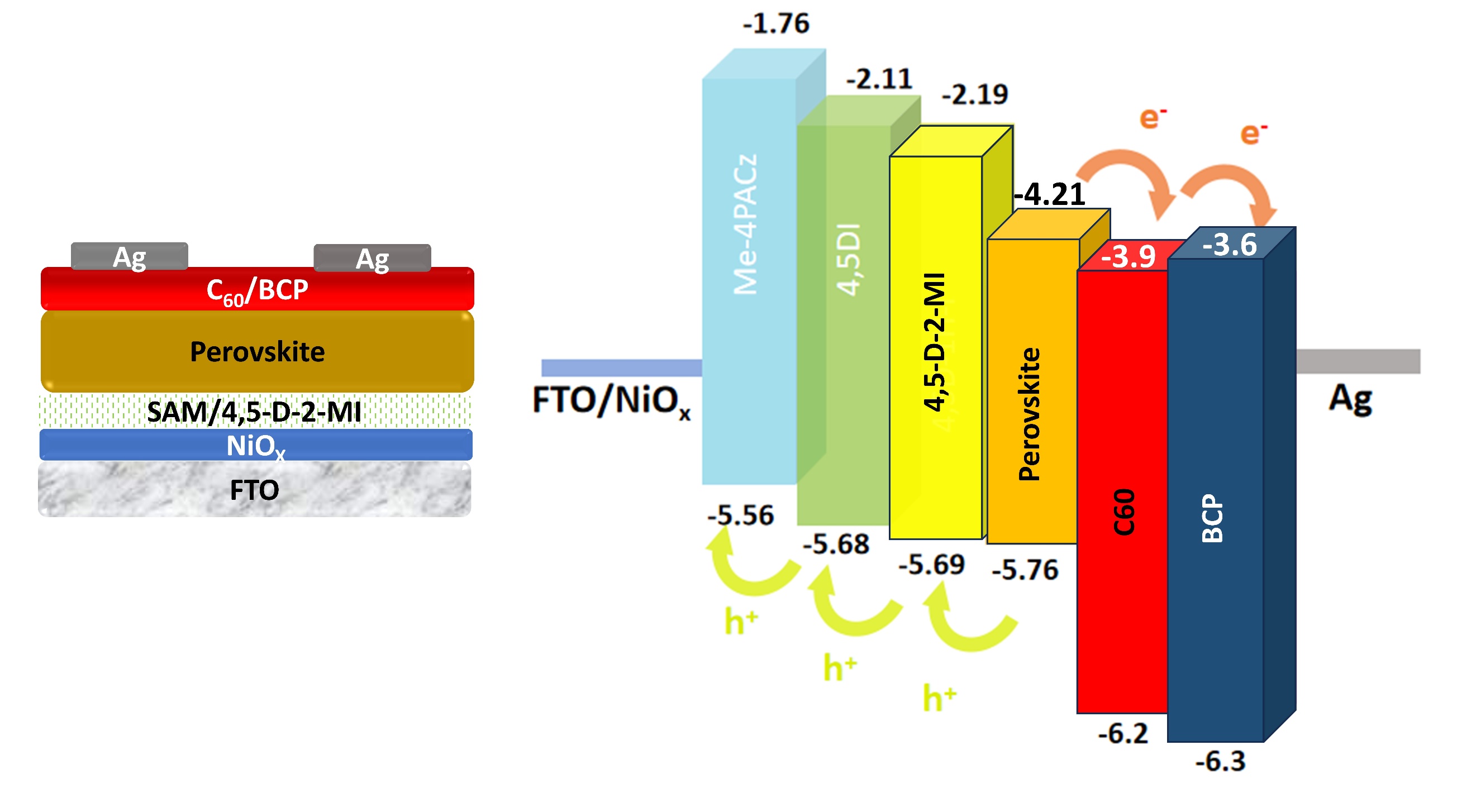
**

**Figure S12**. Targe device architecture and energy level diagram.

**
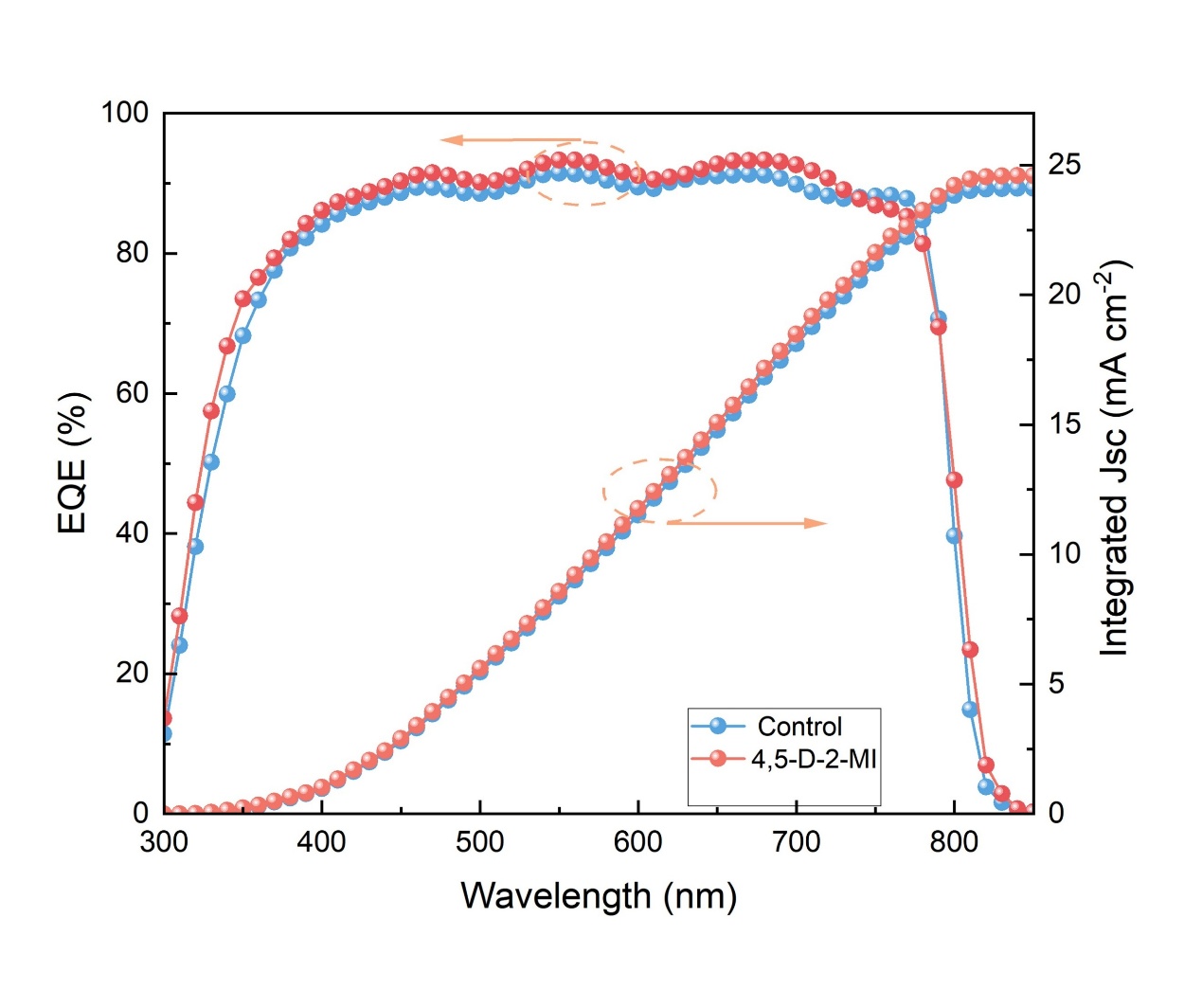
**

**Figure S13**. EQE curves and integrated J_SC_ of control and target device.

**
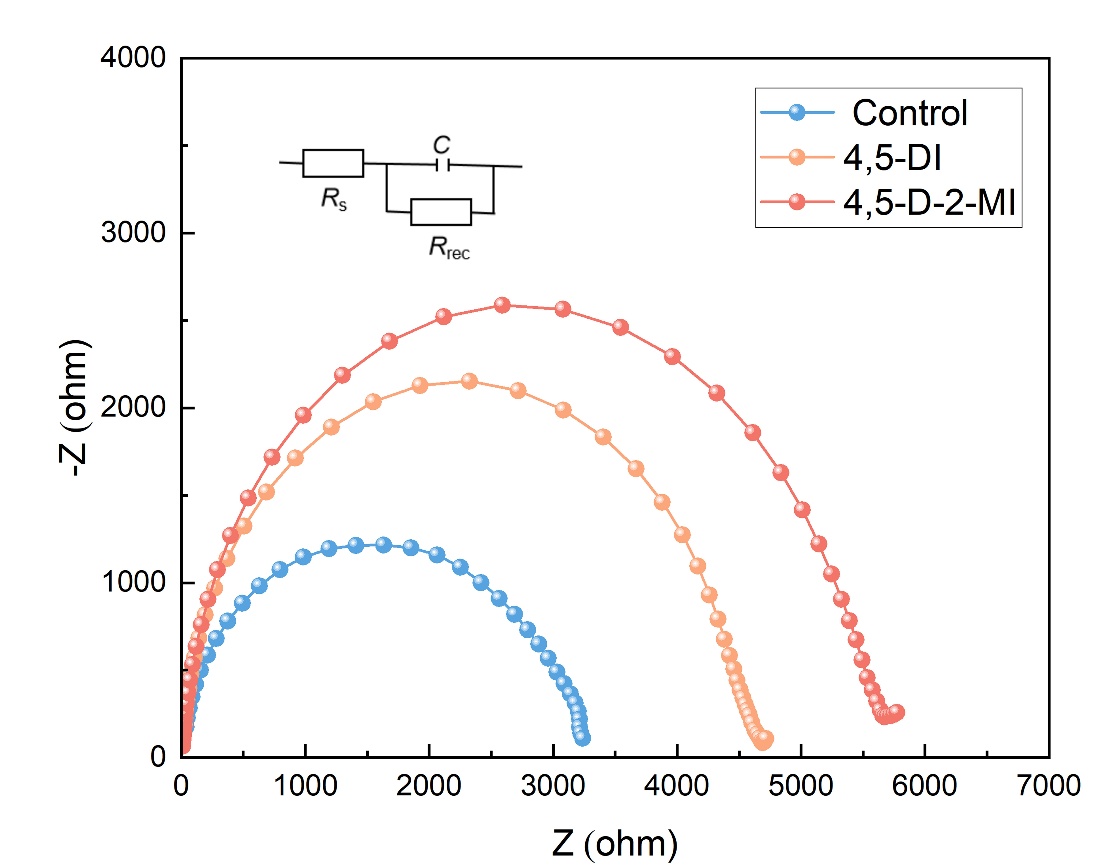
**

**Figure S14.** Nyquist plots of control, 4,5-DI- and 4,55-D-2-MI-based devices.

**
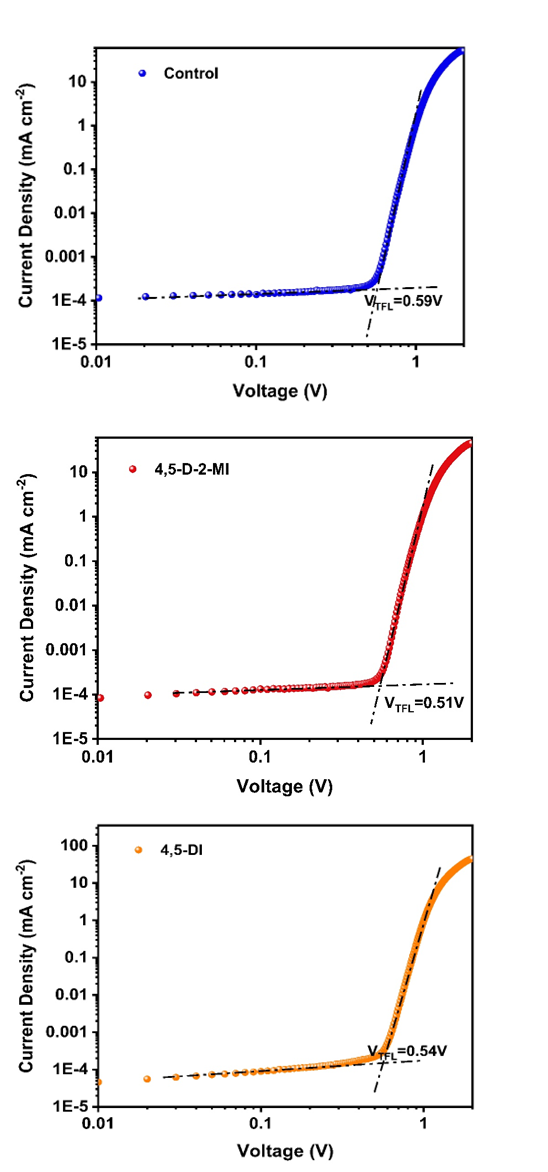
**

**Figure S15**. SCLC analysis of control, 4,5-DI- and 4,55-D-2-MI-based devices.


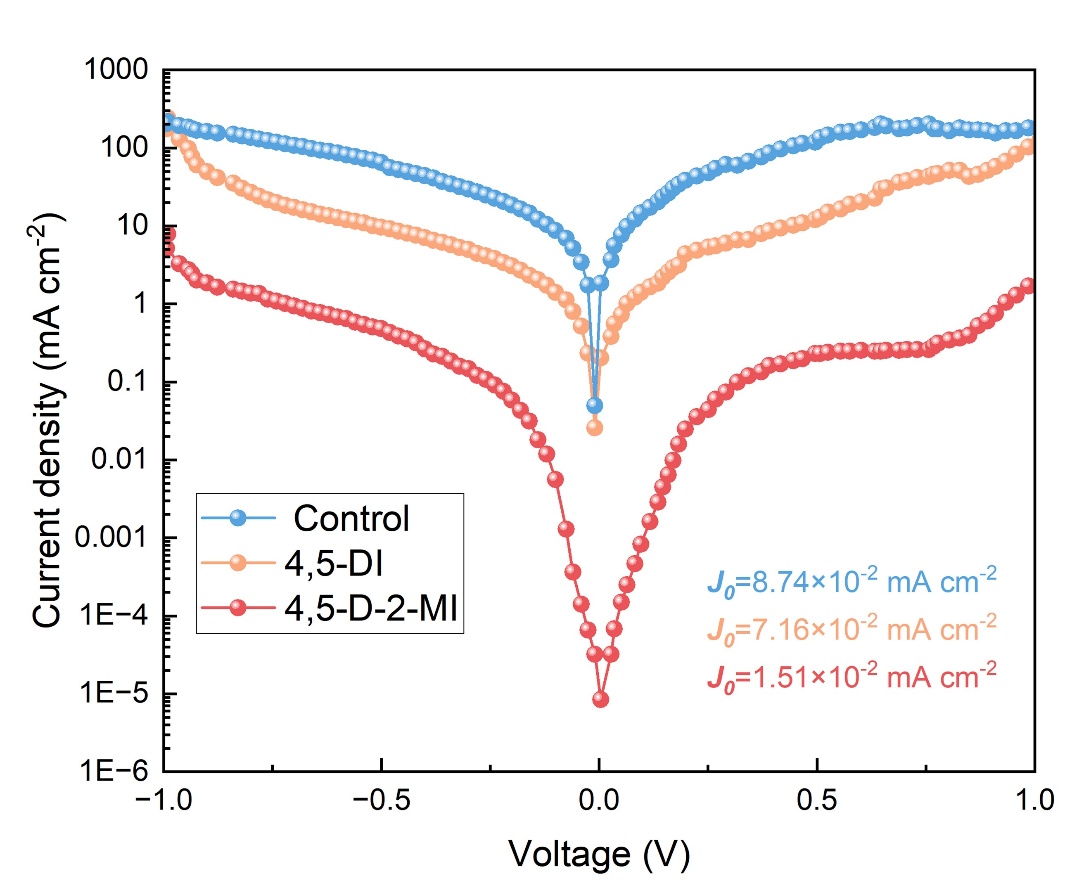


**Figure S16**. JV characteristics under dark conditions showing a decrease in dark current on post-treatment of Me-4PACz via 4,5-DI and 4,5-D-2-MI.

**Supporting Tables**

**Table S1.** The fitted carrier lifetime of perovskite films obtained from the TRPL measurements.

|  | *τ*_1_ (ns) | *τ*_2_ (ns) | *τ*_ave_ (ns) |
| --- | --- | --- | --- |
| Control | 93.42 | 991.63 | 925.16 |
| 4,5-DI | 105.88 | 1553.37 | 1409.98 |
| 4,5-D-2-MI | 108.78 | 1643.45 | 1517.66 |

**Note:**  The average weighted lifetime can be obtained by utilizing a specific equation. In this equation $\tau$ave$=\frac{\sum A_{i}\tau_{i}^{2}}{\sum A_{i}\tau_{i}}$,, there are parameters like A_1_ and A_2_ which stand for the amplitude fraction corresponding to each decay component respectively. While, the symbols τ_1_ and τ_2_ denote the time constant of the two different types of decay. Through this equation, we are able to extract the average weighted lifetime.

**Table S2.** The fitted carrier lifetime of quenched perovskite films obtained from the fs-TAS measurements

|  | *τ*_1_ (ps) | *τ*_2_ (ps) | *τ*_3_ (ps) |
| --- | --- | --- | --- |
| Control/PCBM | 19.2 | 144.6 | 1712.8 |
| 4,5-DI/PCBM | 13.6 | 119.2 | 1556.3 |
| 4,5-D-2-MI/PCBM | 11.5 | 97.4 | 1489.1 |

**Table S3.** Fitted values of EIS measurement from the equivalent circuit components.

|  | R_S_ (Ω) | R_rec_ (Ω) | C (nF) |
| --- | --- | --- | --- |
| Control | 24.4 | 3347 | 31.4 |
| 4,5-DI | 27.6 | 7712 | 26.1 |
| 4,5-D-2-MI | 29.1 | 5756 | 22.7 |

**References**

[1] a)J. Hafner, *J. Comput. Chem.* **2008**, 29, 2044; b)G. Kresse, D. Joubert, *Phys. Rev. B* **1999**, 59, 1758.

[2] a)M. Ernzerhof, G. E. Scuseria, *J. Chem. Phys.* **1999**, 110, 5029; b)R. Van Leeuwen, E. Baerends, *Phys. Rev. A* **1994**, 49, 2421.

[3] J. Moellmann, S. Grimme, *J. Phys. Chem. C* **2014**, 118, 7615.

[4] P. Wisesa, K. A. McGill, T. Mueller, *Phys. Rev. B* **2016**, 93, 155109.

[5] C. H. Suresh, G. S. Remya, P. K. Anjalikrishna, *Wiley Interdiscip. Rev.: Comput. Mol. Sci.* **2022**, 12, e1601.

[6] C. W. Bauschlicher Jr, H. Partridge, *Chem. Phys. Lett.* **1995**, 240, 533.

[7] M. P. Andersson, P. Uvdal, *J. Phys. Chem. A* **2005**, 109, 2937.
